# Retrospective-Prospective Observational Study of Italian Patients Treated in Melanoma Adjuvant Cohort MAP–MADAM (Maximing ADjuvAnt MAP): Interim Analysis

**DOI:** 10.3390/cancers16234072

**Published:** 2024-12-05

**Authors:** Francesca Consoli, Marco Tucci, Jacopo Pigozzo, Ester Simeone, Francesco Spagnolo, Teresa Troiani, Francesca Morgese, Michele Del Vecchio, Barbara Melotti, Maria Chiara Tronconi, Maria Francesca Morelli, Federica Grosso, Barbara Merelli, Ilaria Marcon, Diletta Valsecchi, Pietro Quaglino

**Affiliations:** 1 Medical Oncology, ASST Spedali Civili, 25123 Brescia, Italy; francesca.consoli@libero.it; 2 Medical Oncology Unit, University of Bari Aldo Moro, Policlinico Hospital of Bari, 70124 Bari, Italy; marco.tucci@uniba.it; 3 Veneto Institute of Oncology IOV-IRCCS, 35128 Padova, Italy; jacopo.pigozzo@iov.veneto.it; 4 Unit of Melanoma, Cancer Immunotherapy and Innovative Therapy, National Cancer Institute G. Pascale, 80131 Naples, Italy; ester.simeone@gmail.com; 5 Department of Medical Oncology, Oncologia Medica 2, IRCCS Ospedale Policlinico San Martino, 16132 Genoa, Italy; francesco.spagnolo@hsanmartino.it; 6 Department of Surgical Sciences and Integrated Diagnostics (DISC), Plastic Surgery, University of Genova, 16132 Genoa, Italy; 7 Medical Oncology Unit, Department of Precision Medicine, University of Campania “Luigi Vanvitelli”, 80123 Naples, Italy; teresa.troiani@unicampania.it; 8 Oncology Clinic, Polytechnic University of Marche, United Hospitals of Ancona, 60126 Ancona, Italy; francescamorgese85@gmail.com; 9 S.S. Oncologia Medica Melanomi, S.C. Oncologia Medica 1, Dipartimento di Oncologia Medica ed Ematologia, Fondazione IRCCS Istituto Nazionale dei Tumori, 20133 Milan, Italy; michele.delvecchio@istitutotumori.mi.it; 10 Divisione di Oncologia Medica, IRCCS Azienda Ospedaliero-Universitaria di Bologna, 40138 Bologna, Italy; barbara.melotti@aosp.bo.it; 11 IRCCS Humanitas Research Hospital, Rozzano, 20089 Milan, Italy; maria_chiara.tronconi@cancercenter.humanitas.it; 12 Oncologia e Oncologia Dermatologica, Istituto Dermopatico dell’Immacolata IDI-IRCCS, 00167 Rome, Italy; m.morelli@idi.it; 13 Mesothelioma and Rare Cancers Unit, Azienda Ospedaliera SS Antonio e Biagio e Cesare Arrigo, 15121 Alessandria, Italy; federica.grosso@ospedale.al.it; 14 Unit of Medical Oncology, ASST Papa Giovanni XXIII, 24127 Bergamo, Italy; bmerelli@asst-pg23.it; 15 Novartis Farma S.p.A, 20154 Milan, Italy; ilaria_gioia.marcon@novartis.com (I.M.); diletta.valsecchi@novartis.com (D.V.); 16 Dermatology Clinic, Department of Medical Sciences, University of Turin, 10126 Turin, Italy

**Keywords:** dabrafenib plus trametinib, melanoma, stage III, relapse-free survival, adjuvant

## Abstract

**Background/Objective**: Dabrafenib and trametinib (D + T) have been approved for the treatment of stage III melanoma with BRAF V600E V600K mutations in an adjuvant setting, based on the results from the COMBI-AD trial. To provide early access to this combination therapy prior to its commercial availability in Italy, a Managed Access Program (MAP) was run in Italy from June 2018 to December 2019. **Methods**: The MADAM (Maximing ADjuvAnt MAP) study is an Italian retrospective–prospective observational study that included patients who received at least one dose of D + T through the MAP. The primary endpoints were relapse-free survival (RFS) and overall survival (OS). **Results**: This interim analysis presents findings after the first 24 months of follow-up. A total of 310 patients were included in the study; 240 completed the 12-month treatment with D + T, while 70 discontinued the combination. RFS rates were 93.2% at 12 months and 80.2% at 24 months. The median RFS was not reached for the overall population or any subgroups. Similarly, the median OS was not reached, with OS rates of 96.4% at 12 months and 92.5% at 24 months. **Conclusions**: D + T achieved an RFS benefit, with effects sustained beyond the treatment period, indicating positive outcomes in this patient population.

## Simple Summary

Following surgery, dabrafenib and trametinib have been approved for the treatment of stage III melanoma with a B-RAF gene mutation. A retrospective–prospective observational trial conducted in Italy, known as MADAM (Maximizing ADjuvAnt MAP), included patients who had received at least one dose of trametinib plus dabrafenib. Following the first 24 months of follow-up, this analysis was the first. The trial involved 310 patients in total, of whom 240 completed the 12-month course of treatment, while 70 discontinued therapy. At 24 months, the majority of patients (80.2%) were alive and had not experienced disease relapse. The combination of trametinib and dabrafenib appears to benefit patients by lowering the rate of relapse.

## 1. Introduction

Melanoma is an aggressive skin cancer with a dismal prognosis at advanced stages and a median survival time of 7–9 months in metastatic settings [1,2,3]. The development of molecularly targeted treatments and immune checkpoint blockade drugs has significantly improved the prognosis for advanced cases [4,5,6]. Currently, certain patient cohorts have median survival rates exceeding 4–5 years [7]. In Italy, an estimated 12,700 new diagnoses of melanoma of the skin are expected in 2023, with a five-year survival rate of 90% [8].

Surgical resection of cutaneous melanoma is the standard initial treatment for early-stage disease and is represented by excision of the primary and sentinel lymph node biopsy for T1b to T4 melanomas [9,10,11]. Many treatments, including dabrafenib plus trametinib (D + T), were transitioned into the resectable disease setting [9,12,13,14,15] as postoperative (adjuvant) approaches after a successful use in the advanced-stage, unresectable melanoma, where they considerably improved patient survival [16,17].

The efficacy and safety of D + T (dabrafenib 150 mg twice daily + trametinib 2 mg once daily) in an adjuvant setting (12-month treatment) was assessed in the phase III, randomized, double-blind, placebo-controlled trial COMBI-AD involving 870 patients with stage III melanoma with BRAF V600E or V600K mutations and pathologic involvement of regional lymph node(s). It should be noted that, in the COMBI-AD trial, complete node dissection was mandatory in patients enrolled and the AJCC classification version used was 7.0 [18], while in following trials it was the AJCC version 8.0 [19]; in this trial, in the D + T group, the estimated relapse-free survival (RFS) was 88% (95% confidence interval CI, 85–91) at 12 months, 67% (95%CI 62–72%) at 24 months [20] and 58% (95%CI, 55–64) at 36 months compared, respectively, to 56%, 44%, and 39% in the placebo group (hazard ratio [HR] for relapse or death, 0.47; 95%CI, 0.39–0.58; *p* < 0.001) [21]. Long-term data after a median follow-up of 60 months showed a 5-year RFS rate of 52% (95%CI, 48–58%) with D + T vs 36% (95%CI, 32–41%) with placebo [22]. After nearly 10 years of follow-up, the efficacy of adjuvant therapy with dabrafenib plus trametinib was confirmed, demonstrating significantly improved RFS and distant metastasis-free survival compared to placebo in patients with resected stage III melanoma (RFS rate, with hazard ratio for relapse or death 0.52 (95% CI, 0.43 to 0.63) and hazard ratio for distant metastasis or death, 0.56 (95% CI, 0.44 to 0.71)) [23]. Pyrexia was the most frequent side effect of the combination [24,25] and, in the COMBI-AD trial, led to discontinuation in 9% of patients under D + T treatment [21]. The phase IIIb COMBI-A Plus trial was designed to specifically evaluate whether an adapted pyrexia management algorithm [26] could reduce serious pyrexia-related adverse outcomes, thereby minimizing interruptions to this beneficial treatment. In the trial, D + T was interrupted immediately at the onset of pyrexia (≥38 °C) and restarted at the same dose upon improvement of symptoms if patients remained symptom free (temperature < 38 °C) for at least 24 h, thus decreasing the discontinuation rate for pyrexia (2.4%) and maintaining the clinical benefit of treatment [27]. In the COMBI-A Plus trial, RFS at 12 months showed an increase compared to previous trials (91.3%).

Based on efficacy and safety results from the COMBI-AD phase III trial, in 2018 the Food Drug Administration and the European Medicine Agency approved D + T for the adjuvant treatment (1-year treatment) of patients with melanoma harboring BRAF V600E or V600K mutations, as detected by an approved test, and with involvement of lymph node(s), following complete resection.

The Managed Access Program (MAP) was run in Italy from June 2018 to December 2019 to provide D + T in adjuvant settings to patients with BRAF V600 stage III melanoma following complete tumor resection and without alternative treatment options; roughly 550 patients were involved.

Despite the substantial insights gained from pivotal trials, real-world data on D + T effectiveness in stage III melanoma remain limited. Therefore, it is important that the clinical experience of the Italian MAP was treasured in the MADAM study. This report presents an interim analysis 24 months from the initiation of the study.

## 2. Materials and Methods

### 2.1. Study Design

This retrospective–prospective observational study, conducted in Italy, focused on patients enrolled in the melanoma adjuvant Managed Access Program (MAP) between June 2018 and December 2019. The Maximing ADjuvAnt MAP (MADAM) study involved a review of medical records and ongoing monitoring of patients to assess the effectiveness of Dabrafenib plus Trametinib (D + T) in real-world clinical settings. Safety data were not included in the analysis. Information on subsequent therapies, surgeries, and medical procedures over a 5-year period was collected. To be eligible, patients needed to be ≥18 years of age, have a histologically confirmed stage III BRAF V600 mutation-positive cutaneous melanoma, received at least one dose of D + T in the melanoma adjuvant MAP, and completed/discontinued the program for any reason. Patients were asked to sign informed consent form prior to any data collection; in case of deceased patients, informed consent was not required as per local regulations. Ethical approval for the study was secured from the Ethics Committees of all participating centers. The process was coordinated by the Comitato Etico Interaziendale—AOU Città della Salute e della Scienza di Torino—AO Ordine Mauriziano di Torino—ASL Città di Torino, with their initial approval granted on 29 April 2021 (Protocol No. 0047565, ID No. 74/2021). The study adhered to all relevant local laws and principles outlined in the Helsinki Declaration.

### 2.2. Study Procedures

Patients’ data were collected every 6 months for 5 years starting from completion/discontinuation of adjuvant D + T treatment for any reason (e.g., completion of the suggested 12-month treatment period, tumor recurrence, unacceptable toxicity, etc.).

### 2.3. Endpoints

The primary endpoints were relapse-free survival (RFS) and overall survival (OS). RFS was the time, in months, from D + T initiation to disease relapse or death from any cause; OS was the time, in months, from D + T initiation to death from any cause. Both functions were estimated using the Kaplan–Meier product-limit method. The secondary endpoints were local or systemic treatment for melanoma after any relapse/progression (local and/or systemic) or discontinuation of previous treatment due to other causes, reason for treatment switch, and the definition of patterns of relapse/progression. These analyses were repeated comparing patients who completed the 12-month D + T treatment and who prematurely discontinued the adjuvant combination. Within the latter group, the analysis was further stratified to distinguish between patients who discontinued due to relapse versus those who discontinued for reasons other than relapse.

### 2.4. Statistical Analysis

No statistical sample size calculation was performed. Given that approximately 550 Italian patients were eligible for the adjuvant melanoma MAP, and considering the distribution of study sites, available resources, and patient willingness to participate, a total of 387 patients were enrolled in this study. Statistical analyses were descriptive for all endpoints. Continuous data were summarized by mean, standard deviation (SD), median, first and third quartiles, minimum, and maximum; categorical data were presented by absolute and relative frequencies (*n* and %) or contingency tables. Statistical analysis was performed with SAS^®^ release 9.4 (SAS Institute, Inc., Cary, NC, USA).

The Kaplan–Meier product-limit method was applied to compute estimates of the median values of RFS and OS with the relative 95% CI. Kaplan–Meier curves were plotted, and the Wilcoxon test was performed to compare curves of the subgroups.

## 3. Results

**Figure 1:**
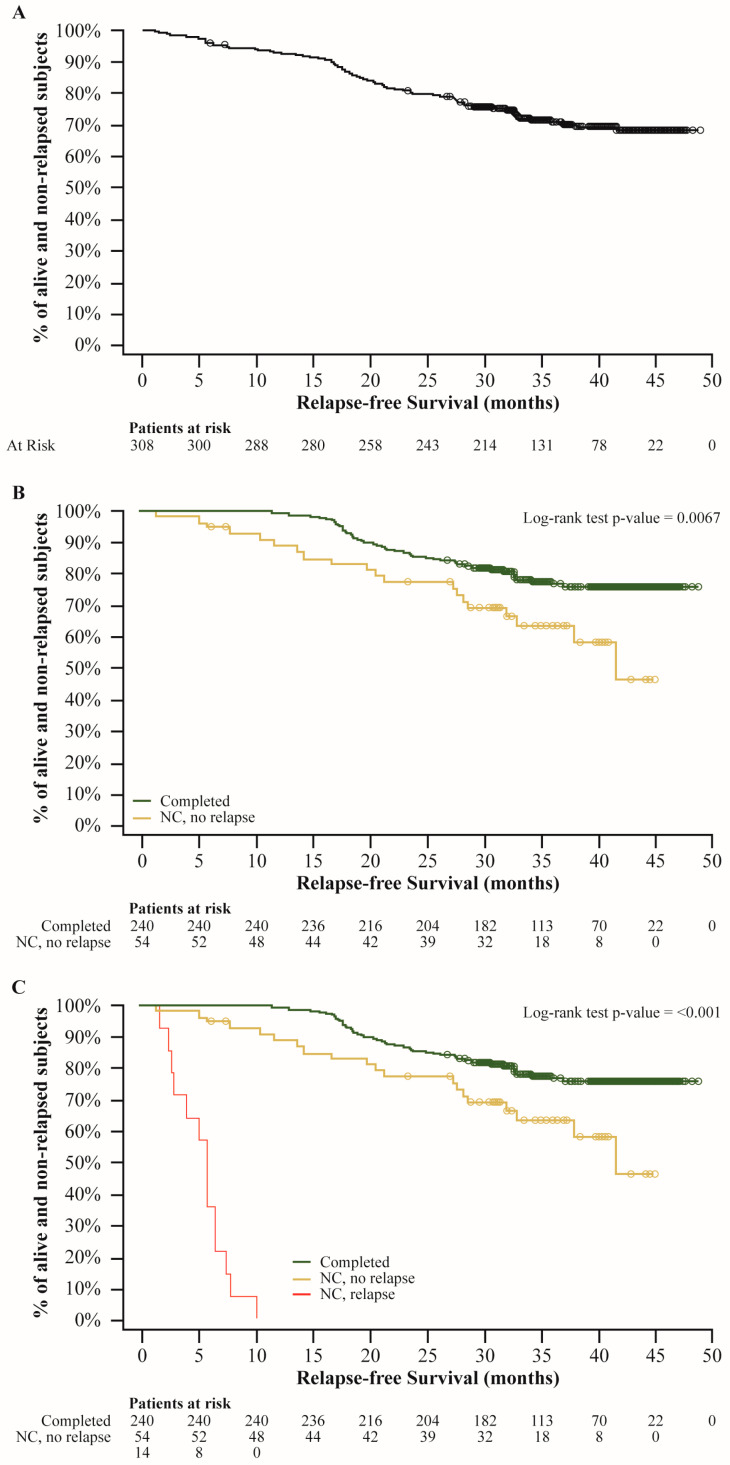
Relapse-free survival estimates of the entire cohort (**A**), according to the completion of 12-month treatment (**B**), and according to reason for discontinuation (**C**). In all graphs, dots represent censors; log-rank test was used instead of Wilcoxon test as specified in the SAP to give equal weight to all time points.

In this interim analysis, 310 patients were included after a median follow-up 35.4 months from D + T initiation; 240 patients completed the 12-month treatment, while 70 (22.5%) discontinued the combination [14 for relapse, 33 for drug-related toxicities, 5 for unrelated toxicities, 18 for other or missed reasons]. The median age was 54.0 years (interquartile range IQR 46.0–65.0), with 58.7% being males; 52.2% of patients had at least one concomitant or past comorbidity. A total of 41.6% of patients had stage IIIC melanoma (Table 1); BRAF mutation V600E was present in 87.1% and V600K in 8% of tumors, median RFS was not reached overall (Figure 1A) and among patients who completed the 12 months treatment, while for patients who did not complete treatment, adjuvant combination was 37.9 months (95%CI, 20.44-NE, *p* < 0.0001) (Figure 1B). Among patients who did not complete the treatment for reasons other than relapse, median RFS was 41.56 months (95%CI, 32.89-NE), while in patients who discontinued for relapse, median RFS was 5.55 months (95%CI, 2.50–6.24) (Figure 1C).

RFS rates were 93.2% at 12 months, 86.9% at 18 months, 80.2% at 24 months, and 70.9% at 36 months in the entire cohort. At 24 months, the RFS rate was 85.4% in the group who completed the treatment and 77.1% in the group of patients who did not complete the treatment; among patients who discontinued for reasons other than relapse, it was 77.1%. RFS rates at 12, 24, and 36 months were summarized in Supplementary Table S1.

**Figure 2:**
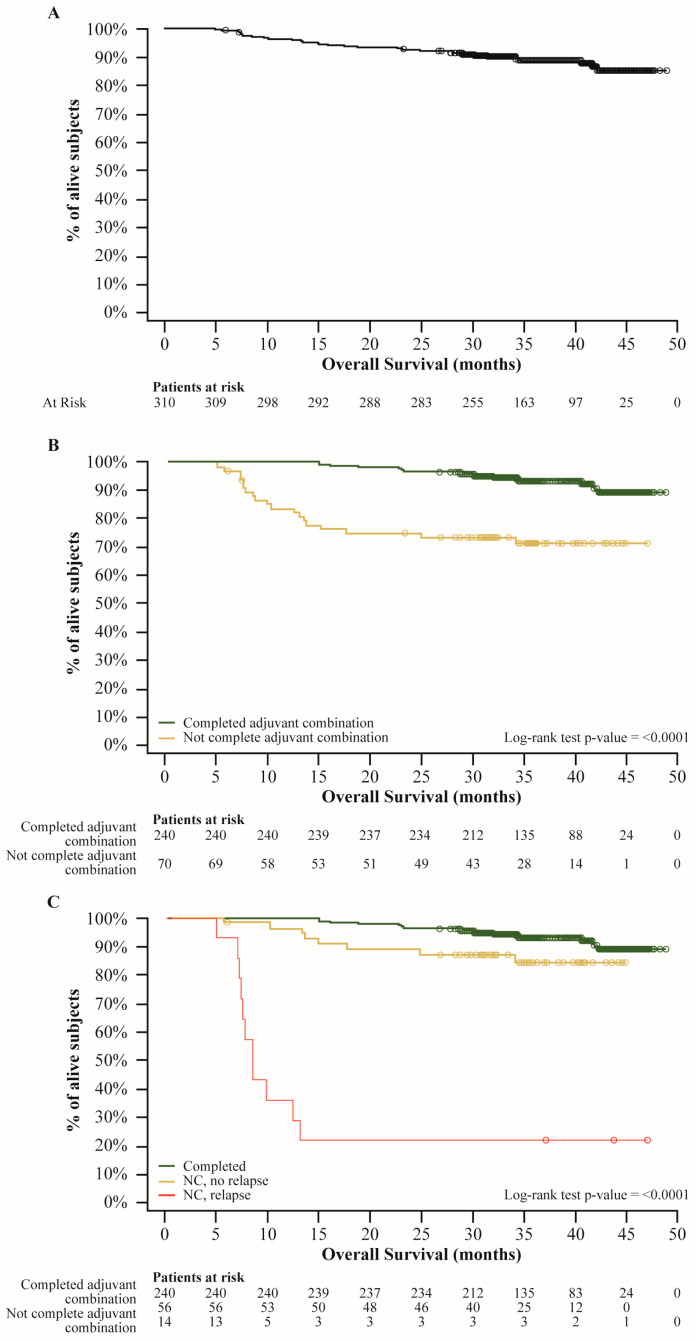
Overall survival estimates of the entire cohort (**A**), according to the completion of 12-month treatment (**B**), and according to reason for discontinuation (**C**). In all graphs, dots represent censors; log-rank test was used instead of Wilcoxon test as specified in the SAP to give equal weight to all time points.

At the time of interim analysis, 35 patients died (33 for melanoma, one for SARS-CoV-2 infection, and one for acute cardiac event). Median OS were not reached in the overall population and subgroups (Figure 2) and the OS rate at 12 months was 96.4%, at 24 months was 92.6%, and 88.9% at 36 months.

Among patients who discontinued for reasons other than relapse, median OS was not reached, while in patients who had relapse, the median OS was 8.44 months (95% CI 7.20, 13.04). OS rates for each group were summarized in Supplementary Table S2.

**Figure 3:**
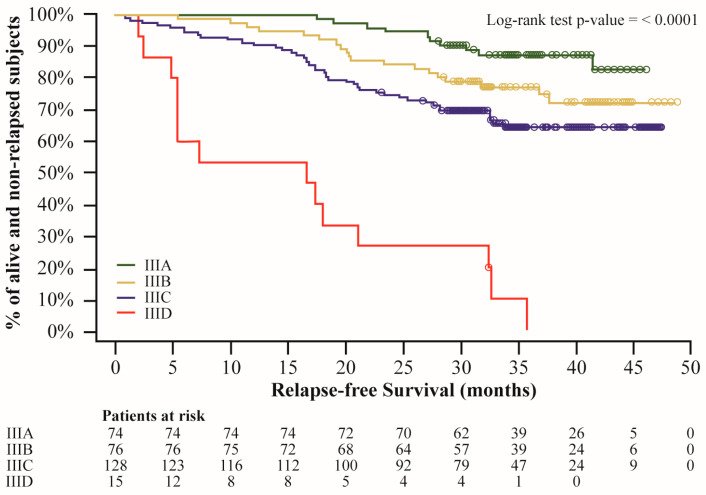
Relapse-free survival estimates according to AJCC v.8. Dots represent censors; log-rank test was used instead of Wilcoxon test as specified in the SAP to give equal weight to all time points.

Stratifying patients according to American Joint Committee on Cancer staging system version 8 (AJCCv8) stage at the time of the start of combination therapy with D + T, median RFS was not reached in IIIA, IIIB, and IIIC subgroups, while in patients with IIID melanoma, RFS was 16.8 months (95%CI, 4.9–21.2) (Figure 3 and Supplementary Table S3).

**Figure 4:**
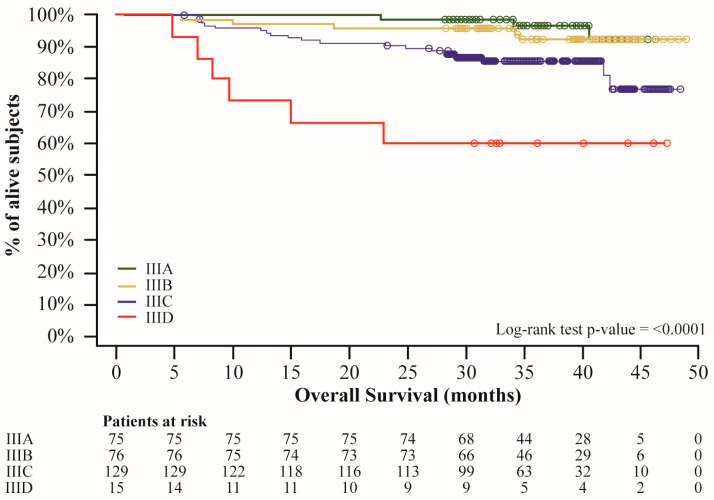
Overall survival estimates according to AJCC v.8. Dots represent censors; log-rank test was used instead of Wilcoxon test as specified in the SAP to give equal weight to all time points.

Median OS was not reached in any subgroups (Figure 4) and OS rates were summarized in Supplementary Table S4.

Overall, the median duration of exposure to D + T was 366.0 days (IQR 349.0–371.0); the median duration was 367 days (IQR 362.0–373.0) in patients who completed adjuvant treatment and 166.5 days (IQR 92.0–318.0) in those who discontinued.

Eighty-six patients (27.7%) had at least one relapse: 53 among patients who completed the treatment and 33 among patients who discontinued. A systemic relapse was reported, respectively, in 38 patients and in 26 patients, with the most frequent metastasis localizations in brain, lung, and lymph nodes. A local relapse, mainly cutaneous, was described, respectively, in 22 and 11 (33.3%) patients (Table 2).

## 4. Discussion

This interim analysis of the MADAM study demonstrated that D + T significantly improved relapse-free survival (RFS), particularly in patients who completed the full 12-month adjuvant treatment as expected. These findings confirm that D + T adjuvant therapy is also effective for melanoma patients in a real-world clinical setting.

Our findings are consistent with the results of the phase III (COMBI-AD) and phase IIIb (COMBI-A Plus) clinical trials, as well as with previously reported real-world experiences. In clinical trials, adjuvant therapy with D + T significantly lowered the recurrence risk, with RFS at 12 months of 88% in the COMBI-AD trial [21] and 91.8% in COMBI-A Plus [27]. The percentage of patients who did not complete the 12-month treatment was similar in our cohort and COMBI-A Plus trial (respectively, 22.5% and 23.2%). The discontinuation rate due to toxicity was 26% in the COMBI-AD trial and 15.4% in the COMBI-A Plus trial. In the latter, an algorithm for managing pyrexia was introduced, which is currently utilized in clinical practice as part of the MAP program. The MADAM study reflected the advantages of this algorithm, showing a discontinuation rate for toxicity of 12.5%.

In a real-world setting, despite the limited data, primarily from retrospective studies, the evidence from various countries has been consistent and supports the findings from clinical trials regarding the effectiveness and tolerability of adjuvant D + T therapy. An observational study collecting data from 65 patients in Spain (DESCRIBE AD) reported a 12-month RFS of 95.3% [28]. A cohort from Germany, Austria, and Switzerland (*n* = 195) showed a 12-month RFS rate of 86.5%, with 96.9% of patients alive during the median follow-up of 17 months [29]. Our data, derived from a larger patient cohort, demonstrated similar RFS rates, aligning with these previous findings, with a 12-month RFS of 93.2%. Patients who discontinued for reasons other than relapse showed progression or relapse prior to those who completed the 12-month treatment; therefore, 12-month completion seemed to achieve the maximal benefit from D + T combination (*p* < 0.0001).

According to AJCCv8 stage [19], at D + T initiation, in our cohort a relevant portion of patients had a disease at stage IIIA (24.19%). This is noteworthy because stage IIIA patients with micrometastases < 1 mm in the sentinel lymph node were excluded from clinical trials [30,31].

At the time of this analysis, median OS estimates were not reached, both in the overall population and in subgroups. However, high 24-month OS rates confirmed D + T effectiveness, as previously reported in clinical trials [21,27]; a longer follow-up would better describe the D + T combination impact on survival.

The main limitations of this study are attributable to its observational nature and may involve missing or incomplete data.

## 5. Conclusions

This interim analysis of the MADAM study described the clinical benefits achieved in an adjuvant setting by using D + T in patients with stage III melanoma with BRAF V600 mutation in the real world. D + T combination guaranteed an important RFS in most patients, sustained after the end of treatment. These data confirmed the effectiveness of D + T in this setting compared to phase III trials (COMBI-AD and COMBI-A Plus). The results consolidated the effectiveness in terms of RFS in non-selected patients, in all stage III subpopulations, including IIIA; among patients who completed the 12-month treatment, an even further benefit was observed.

Patients stopping treatment for reasons other than relapse should be monitored more closely, as their risk of relapse could be higher.

## Figures and Tables

**Table 1:** Baseline and clinical characteristics.

	Cohort (*n* = 310)	Completed Adjuvant Combination (*n* = 240)	Not Completed Adjuvant Combination (*n* = 70)
Age (years), median (IQR)	54.0 (46.0–65.0)	53.0 (46.0–63.5)	58.0 (48.0–71.0)
Female, *n* (%)	128 (41.3)	97 (40.4)	31 (44.3)
Time from initial diagnosis to the D + T combination start date (months), median (IQR)	5.5 (4.3–7.1)	5.5 (4.4–7.3)	5.0 (3.8–6.6)
AJCC 8 Stage at time of start of combination therapy with D + T			
III	15 (4.8)	13 (5.4)	2 (2.8)
IIIA	75 (24.2)	58 (24.1)	17 (24.3)
IIIB	76 (24.5)	63 (26.2)	13 (18.5)
IIIC	129 (41.6)	98 (40.8)	31 (44.3)
IIID	15 (4.8)	8 (3.3)	7 (10.0)
ECOG PS, *n* (%)			
0	183 (95.8)	144 (96.0)	39 (95.1)
1	7 (3.7)	5 (3.3)	2 (4.9)
2	1 (0.5)	1 (0.7)	0

**Table 2:** Summary of relapse/progression by subgroup.

	Completed Adjuvant Combination (*n* = 240)	Not Completed Adjuvant Combination (*n* = 70)
Number of patients with at least one relapse, *n* (%)	53 (22.1)	33 (47.1)
AJCC 8 stage at start of combo for patients with at least one relapse, *n* (%) *		
III	1 (1.9)	0
IIIA	9 (17.0)	2 (6.1)
IIIB	11 (20.7)	6 (18.2)
IIIC	25 (47.2)	18 (54.5)
IIID	7 (13.2)	7 (21.2)
Number of patients with at least one systemic relapse, *n* (%) *	38 (71.7)	26 (78.8)
Localization, *n* (%) a		
Brain	12 (21.4)	8 (17.8)
Visceral, organs involved	32 (57.1)	26 (57.8)
Lungs	8	9
Liver	6	6
Adrenal glands	0	1
Gastrointestinal locations	0	0
Kidney	1	0
Bladder	0	1
Lymph nodes	13	4
Soft tissue	1	1
Other	3	4
Non-visceral, organs involved	12 (21.4)	11 (24.4)
Cutaneous-Subcutaneous distant from primary tumor	6	4
Bones	3	6
Other	3	1
Number of patients with at least one local relapse, *n* (%) *	22 (41.5)	11 (33.3)
Localization, *n* (%) a		
Cutaneous	16 (66.7)	9 (75.0)
Regional lymph nodes	8 (33.3)	3 (25.0)

* percentage refers to the group of patients who showed at least one relapse (53 patients completing the adjuvant combination and 70 patients not completing the adjuvant combination); a percentage refers to the total number of relapse localization.

## Data Availability

The data that support the findings of this study are available from the corresponding author upon reasonable request.

## Supplementary Materials

The following supporting information can be downloaded at: https://www.mdpi.com/article/10.3390/cancers16234072/s1, Supplementary Table S1: Relapse-free survival rates in the entire cohort and according to adjuvant 12-month treatment completion; Supplementary Table S2: Overall survival rates in the entire cohort and according to adjuvant 12-month treatment completion; Supplementary Table S3: Relapse-free survival rates according to AJCCv8 stage subgroups; Supplementary Table S4: Overall survival rates according to AJCCv8 stage subgroups.
