# Supplementary material for: Retrospective-Prospective Observational Study of Italian Patients Treated in Melanoma Adjuvant Cohort MAP–MADAM (Maximing ADjuvAnt MAP): Interim Analysis
Source: Cancers (Basel). 2024 Dec 5;16(23):4072. doi: 10.3390/cancers16234072 (PMC11640251; doi:10.3390/cancers16234072)
Supplement: Supplementary file 1 [file cancers-16-04072-s001.zip › cancers-3276976-supplementary.pdf]

Supplementary Table S1. Relapse free survival rates in the entire cohort and according to adjuvant 12-month treatment completion .

	Cohort (n=310)	Completed adjuvant combination (n=240)	Not completed adjuvant combination (n=70)	Not completed, no relapse (n=56)	Not completed, relapse (n=14)
**RFS, median (95% CI) months**	NE (NE, NE)	NE (NE, NE) p<0.0001	37.91 (20.44, NE)	41.56 (32.89, NE)	5.55 (2.50, 6.24)
**RFS at 12 months n (%) Rate**	21 (6.8) 93.1%	1 (0.42) 99.6%	20 (29.4) 70.2%	6 (11.1) 88.6%	14 (100) 0%
**RFS at 24 months n (%) Rate**	61 (19.8) 80.2%	35 (14.6) 85.4%	26 (38.2) 61.8%	12 (22.2) 77.1%	14 (100) 0%
**RFS at 36 months n (%) Rate**	85 (27.6) 70.9%	53 (22.1) 76.6%	32 (47.1) 50.4%	18 (33.3) 63.7%	14 (100) 0%

Supplementary Table S2. Overall survival rates in the entire cohort and according to adjuvant 12-month treatment completion.

	**Cohort (n=310)**	**Completed adjuvant combination (n=240)**	**Not completed adjuvant combination (n=70)**	**Not completed, no relapse (n=56)**	**Not completed, relapse (n=14)**
**OS at 12 months n (%) Rate**	11 (3.5)<br>96.4%	0<br>100%	11 (15.7)<br>83.9%	2 (3.6)<br>96.4%	9 (64.3)<br>35.7%
**OS at 24 months n (%) Rate**	23 (7.4)<br>92.6%	6 (2.5)<br>97.5%	17 (24.3)<br>75.7%	6 (10.7)<br>88.9%	11 (78.6)<br>21.4%
**OS at 36 months n (%) Rate**	32 (10.3)<br>88.9%	13 (5.4)<br>93.9%	19 (27.1)<br>71.4%	8 (14.3)<br>84.2%	11 (78.6)<br>21.4%

Supplementary Table S3. Relapse free survival rates according to AJCCv8 stage subgroups.

	**IIIA (n=75)**	**IIIB (n=76)**	**IIIC (n=129)**	**IIID (n=15)**
**RFS, median (95% CI) months**	NE (NE, NE) p<0.0001	NE (NE, NE)	NE (NE, NE)	16.8 (4.9, 21.2)
**RFS at 12 months n (%) Rate**	0 100%	3 (3.9) 96.0%	11 (8.6) 91.3%	7 (46.7) 53.3%
**RFS at 24 months n (%) Rate**	4 (5.4) 94.6%	12 (15.8) 84.2%	32 (25) 75%	11 (73.3) 26.7%
**RFS at 36 months n (%) Rate**	9 (12.2) 87.2%	17 (22.4) 77.4%	43 (33.6) 64.2%	14 (93.3) 0

Supplementary Table S4. Overall survival rates according to AJCCv8 stage subgroups.

	IIIA (n=75)	IIIB (n=76)	IIIC (n=129)	IIID (n=15)
**OS at 12 months**				
**n (%)**	0	2 (2.6)	5 (3.9)	4 (26.7)
**Rate**	100%	97.4%	96.1%	73.3
**OS at 24 months**				
**n (%)**	1 (1.3)	3 (3.9)	12 (9.3)	6 (40)
**Rate**	98.7%	96.1%	90.7%	60%
**OS at 36 months**				
**n (%)**	2 (6.7)	5 (6.6)	18 (13.9)	4 (40)
**Rate**	96.8%	92.3%	85.4%	60%
